# Brain Function Outcomes of Recent and Lifetime Cannabis Use

**DOI:** 10.1001/jamanetworkopen.2024.57069

**Published:** 2025-01-28

**Authors:** Joshua L. Gowin, Jarrod M. Ellingson, Hollis C. Karoly, Peter Manza, J. Megan Ross, Matthew E. Sloan, Jody L. Tanabe, Nora D. Volkow

**Affiliations:** 1 Department of Radiology, University of Colorado Anschutz Medical Campus, Aurora; 2 Department of Psychiatry, University of Colorado Anschutz Medical Campus, Aurora; 3 Department of Psychology, Colorado State University, Fort Collins; 4 Laboratory of NeuroImaging, National Institute on Alcohol Abuse and Alcoholism, Bethesda, Maryland; 5 Addictions Division, Centre for Addiction and Mental Health, Toronto, Ontario, Canada; 6 Department of Pharmacology and Toxicology, University of Toronto, Toronto, Ontario, Canada; 7 Division of Neurosciences and Clinical Translation, Department of Psychiatry, University of Toronto, Toronto, Ontario, Canada; 8 Campbell Family Mental Health Research Institute, Centre for Addiction and Mental Health, Toronto, Ontario, Canada; 9 Department of Psychological Clinical Science, University of Toronto Scarborough, Toronto, Ontario, Canada; 10 Institute of Medical Science, University of Toronto, Toronto, Ontario, Canada; 11 Institute for Mental Health Policy Research, Centre for Addiction and Mental Health, Toronto, Ontario, Canada; 12 National Institute on Drug Abuse, Bethesda, Maryland

## Abstract

**Importance:**

Cannabis use has increased globally, but its effects on brain function are not fully known, highlighting the need to better determine recent and long-term brain activation outcomes of cannabis use.

**Objective:**

To examine the association of lifetime history of heavy cannabis use and recent cannabis use with brain activation across a range of brain functions in a large sample of young adults in the US.

**Design, Setting, and Participants:**

This cross-sectional study used data (2017 release) from the Human Connectome Project (collected between August 2012 and 2015). Young adults (aged 22-36 years) with magnetic resonance imaging (MRI), urine toxicology, and cannabis use data were included in the analysis. Data were analyzed from January 31 to July 30, 2024.

**Exposures:**

History of heavy cannabis use was assessed using the Semi-Structured Assessment for the Genetics of Alcoholism, with variables for lifetime history and diagnosis of cannabis dependence. Individuals were grouped as heavy lifetime cannabis users if they had greater than 1000 uses, as moderate users if they had 10 to 999 uses, and as nonusers if they had fewer than 10 uses. Participants provided urine samples on the day of scanning to assess recent use. Diagnosis of cannabis dependence (per *Diagnostic and Statistical Manual of Mental Disorders, Fourth Edition* criteria) was also included.

**Main Outcomes and Measures:**

Brain activation was assessed during each of the 7 tasks administered during the functional MRI session (working memory, reward, emotion, language, motor, relational assessment, and theory of mind). Mean activation from regions associated with the primary contrast for each task was used. The primary analysis was a linear mixed-effects regression model (one model per task) examining the association of lifetime cannabis and recent cannabis use on the mean brain activation value.

**Results:**

The sample comprised 1003 adults (mean [SD] age, 28.7 [3.7] years; 470 men [46.9%] and 533 women [53.1%]). A total of 63 participants were Asian (6.3%), 137 were Black (13.7%), and 762 were White (76.0%). For lifetime history criteria, 88 participants (8.8%) were classified as heavy cannabis users, 179 (17.8%) as moderate users, and 736 (73.4%) as nonusers. Heavy lifetime use (Cohen *d* = −0.28 [95% CI, −0.50 to −0.06]; false discovery rate corrected *P* = .02) was associated with lower activation on the working memory task. Regions associated with a history of heavy use included the anterior insula, medial prefrontal cortex, and dorsolateral prefrontal cortex. Recent cannabis use was associated with poorer performance and lower brain activation in the working memory and motor tasks, but the associations between recent use and brain activation did not survive false discovery rate correction. No other tasks were associated with lifetime history of heavy use, recent use, or dependence diagnosis.

**Conclusions and Relevance:**

In this study of young adults, lifetime history of heavy cannabis use was associated with lower brain activation during a working memory task. These findings identify negative outcomes associated with heavy lifetime cannabis use and working memory in healthy young adults that may be long lasting.

## Key Points

**Question:**

Are recent cannabis use and lifetime cannabis use associated with differences in brain function during cognitive tasks?

**Findings:**

In this cross-sectional study of 1003 young adults, heavy lifetime cannabis use was associated with lower brain activation during a working memory task; this association remained after removing individuals with recent cannabis use. These results were not explained by differences in demographic variables, age at first cannabis use, alcohol use, or nicotine use.

**Meaning:**

These findings suggest that cannabis use is associated with short- and long-term brain function outcomes, especially during working memory tasks.

## Teaser

This cross-sectional study examines the association of lifetime cannabis use and recent cannabis use with brain function during cognitive tasks among young adults.

## Introduction

As more states and countries have legalized the production and sale of cannabis for recreational and medical use,^1^ there has been an associated increase in the potency of cannabis products,^2^ cannabis use rates,^3,4^ and prevalence of cannabis use disorder.^5^ Greater accessibility of cannabis has also been associated with higher rates of cannabis-related motor vehicle crashes,^6,7^ and frequent cannabis use is associated with increased risk for hyperemesis syndrome^8^ and cardiovascular disease.^9,10^ Despite these negative effects, there is an increasing perception that cannabis is harmless.^11^ Thus, better understanding of recent and long-term effects of cannabis is critical for informing public health policies. Meta-analytic evidence indicates that short-term effects of cannabis include decreases in cognitive performance (eg, episodic verbal memory), but these reductions may not persist after 72 hours of abstinence.^12^ Given the cognitive effects of cannabis and the disruption of the endogenous cannabinoid system by tetrahydrocannabinol (THC),^13,14^ it may be that brain regions with high cannabinoid 1 (CB1) receptor density^15^ might be altered by cannabis. For example, there is evidence that cannabis use among adolescents is negatively associated with the thickness of the left prefrontal cortex (PFC) and right PFC and that the spatial pattern of cannabis-related cortical thinning is related to CB1 receptor density.^16^

Numerous brain imaging studies have examined the effects of cannabis on brain function. For example, relative to nonusers, frequent cannabis users showed a greater response to cannabis cues in the striatum and medial PFC, and activation of these regions correlated with cannabis craving.^17^ There may also be developmental interaction effects.^18^ For example, individuals with cannabis dependence, relative to matched control participants, showed greater functional connectivity density (ie, hyperconnectivity with surrounding regions) in the ventral striatum, and effects were more pronounced in individuals who began cannabis use earlier in life.^19^ Evidence has indicated that cannabis use reduces neural activation related to memory,^20^ executive function,^21,22^ emotion,^23,24^ reward processing,^25^ and social processing,^26^ but most of these previous studies had fewer than 30 participants with cannabis use history.^20^ Furthermore, whereas several efforts have successfully meta-analyzed the cognitive effects of cannabis across multiple domains,^12,27^ few have addressed the effects of cannabis use on brain function across multiple domains. It is also challenging to account for effects on multiple brain regions with an interpretable and clinically meaningful outcome, even though activation patterns of brain regions during tasks are not independent and, instead, are often highly correlated across regions. Evidence from a 2024 study suggests that brain analysis should consider features such as function, architectonics, connectivity, and topography.^28^ Such approaches, however, have seldom been applied to analysis of the effects of cannabis on brain function to help advance knowledge of the influence of history of use or recent use. Such work stands to improve understanding of how cannabis affects neural processing relevant to social, cognitive, and emotional function.

To address these knowledge gaps, we used data from the Human Connectome Project (HCP) for this study. The HCP has data across 7 tasks covering a range of brain functions. It also assesses lifetime cannabis use, cannabis dependence diagnosis (per *Diagnostic and Statistical Manual of Mental Disorders, Fourth Edition* [*DSM-IV*] criteria), and age at first use and uses a urine toxicology screen at the time of scanning to assess for recent cannabis use. These data allowed us to disentangle outcomes associated with a lifetime history of cannabis use from those associated with recent use. The HCP dataset also allowed us to adjust for group differences between individuals with heavy, moderate, and no cannabis use, given that demographic and socioeconomic factors can influence brain function.^29^ We were also able to control for comorbid substance (eg, alcohol or nicotine) use, which is necessary to reduce the likelihood that any observed outcomes of cannabis use are actually attributable to use of other substances. Given that the largest effects of cannabis use are on learning, working memory,^30^ and verbal episodic memory,^12^ we hypothesized that cannabis would be associated with activation during working memory and language tasks, and that this association would be present for recent use and lifetime history of use.

## Methods

Procedures for this cross-sectional study were approved by the Washington University Institutional Review Board. Participants provided written consent. We preregistered our analysis on the Open Science Framework repository.^31^ The study followed the Strengthening the Reporting of Observational Studies in Epidemiology (STROBE) reporting guideline.

### Participants

The HCP study consisted of 1206 adults aged 22 to 37 years,^32^ of whom 1005 had functional magnetic resonance imaging (MRI) data. The goal of the study was to “chart the neural pathways that underlie brain function and behavior.”^32^ Participants were recruited to complete MRI scans at Washington University in Saint Louis, Missouri. The study sampled twins and their nontwin siblings, along with singleton community members.^32^ Sibling status, representing a biological relationship, was based on self-report and genotyping from blood or saliva samples. The study was launched in 2010, and scanning began in August 2012 and concluded in 2015.^33^ For this analysis, we accessed data from the 2017 preprocessed data release. Although we initially planned to examine the language task only, we decided to analyze all 7 functional MRI tasks to broaden our understanding of brain function outcomes of cannabis use.

### Assessment of Cannabis Use

To assess recent use, participants provided urine samples on the day of scanning that were tested for the presence of cannabis metabolites. For analysis, we categorized individuals as having recent use if they had a positive THC result on the Accutest multidrug screen (Jant). A positive result is indicated if urine concentrations of the THC metabolite 11-Nor-Δ9-tetrahydrocannabinol-9-carboxylic acid (THC-COOH) exceed 50 ng/mL. This result typically indicates use in the past 10 days; however, very frequent users may have THC-COOH levels greater than 50 ng/mL for 1 month, or on some occasions several months, post use.^34^

To assess lifetime use, participants completed the Semi-Structured Assessment for the Genetics of Alcoholism (SSAGA).^35^ The interview assessed total lifetime number of cannabis uses on a Likert-rated scale (with response levels of never, 1-5, 6-10, 11-100, 101-999, and >1000). We categorized individuals as nonusers if they had used 10 or fewer times, as moderate users if they had used 11 to 999 times, and as heavy users if they had used 1000 or more times. The lifetime use variable was treated as an ordered factor (nonusers, 0; moderate users, 1; and heavy users, 2).

The SSAGA also assessed a diagnosis of cannabis dependence (lifetime) (per *DSM-IV* criteria) and age at first cannabis use (never or <14, 15-17, 18-20, or >21 years). We treated these as covariates.

### Demographic and Clinical Assessment

Participants completed demographic screening to indicate age, sex, race, income, and education. Race was self-reported as American Indian or Alaska Native, Asian (including Native Hawaiian or Other Pacific Islander), Black or African American (hereinafter, Black), White, multiple races, or race unknown or not reported. These data were collected because sociocultural influences associated with race may also be associated with both cannabis use and brain activation patterns. We did not have sufficient data on ethnicity to analyze. Additional substance use variables were assessed with the SSAGA, including alcohol dependence diagnosis, and the Fagerström Test for Nicotine Dependence.^36^ To generate a single metric each for (1) alcohol use and (2) nicotine use that accounted for quantity and frequency, we created *z* score metrics as described previously.^19^ Episodic verbal memory was assessed with Form A of the Penn Word Memory Test.^37^ For crystallized intelligence, participants completed the National Institutes of Health Toolbox Picture Vocabulary Test,^38^ which assesses vocabulary knowledge and is associated with scholastic success^39^ and the “g” factor of intelligence.^40^

### Brain Imaging Tasks

**Figure 1.**
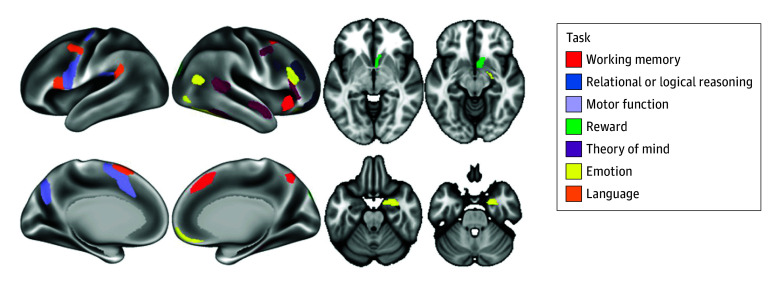
Brain Regions Analyzed for Each Task For each task, we identified the brain regions associated with the primary contrast and used the mean value from all regions associated with that task. This approach was used to simplify the analysis by having a single outcome for each task rather than multiple outcomes, and because the activation levels during the primary contrast were not independent outcomes and were moderately to highly correlated (eg, *r* = 0.5). This approach also allows for an interpretable primary finding that can be investigated through post hoc analysis to identify regions associated with cannabis use. It also accords with theories of brain organization that account for function, architectonics, connectivity, and topography and produce models of brain function that better explain activity patterns. The anterior insula and dorsomedial prefrontal cortex are depicted in red for the working memory task, but the same parcels were also included as part of the relational task model.

Seven tasks were used in this study, aiming to cover a broad range of behavioral processes.^41^ Tasks were chosen based on reliability of neural response and a well-characterized neurocognitive basis. The tasks examined neural response related to emotion, reward, motor function, working memory, language, relational or logical reasoning, and theory of mind or social information processing (task details are provided in the eMethods in Supplement 1). Neural response to the tasks has previously discriminated substance use history.^42^ For each task, we used the primary contrast, and we extracted activation levels from regions positively activated during the task (ie, regions engaged by the task, not regions that were deactivated by the task, such as nodes in the default mode network). Positive activation was defined as having significant activation (2-tailed *P* < .001), with most effect sizes (Cohen *d*) greater than 1.00 for the contrast (the task positive condition minus the control condition).^43^ We used the mean value of the activation levels across the listed regions for each task, so each participant had a single value representing the activation level for each task; the regions used are depicted in Figure 1. We chose to use a single value because (1) it reduced the number of outcomes for analysis, (2) it provided a more clinically interpretable metric, and (3) the activation levels across regions during a given task were not independent.^44^

### Imaging Acquisition and Processing

The HCP investigators used a 3T Connectome Skyra scanner (Siemens) to acquire imaging data. For parameters, see the data acquisition plan published previously.^45^ Preprocessing was conducted as part of the HCP minimally preprocessed dataset with the FMRIB Software Library (Oxford University).^46^

### Statistical Analysis

#### Analytic Approach

Data were analyzed from January 31 to July 30, 2024. To examine participant characteristics, we compared nonusers, moderate users, and heavy users based on race, sex, age, education, income level, urine sample status, cannabis dependence diagnosis, age at first cannabis use, alcohol dependence diagnosis, nicotine dependence score, and alcohol *z* scores indicating quantity or frequency of use. We used the Wilcoxon rank sum test or the Fisher exact test to determine significance levels for the Table, and we report χ^2^ values in the Results. Statistical significance was set at a 2-tailed α ≤ .05.

**Table.** Participant Characteristics by History of Cannabis Use^a^

Characteristic	Nonuser (n = 736)	Moderate user (n = 179)	Heavy user (n = 88)	*P* value
Race				
American Indian or Alaska Native	1 (0.1)	0	1 (1.1)	<.001
Asian (including Native Hawaiian or Other Pacific Islander)	56 (7.6)	6 (3.4)	1 (1.1)	
Black or African American	96 (13.0)	22 (12.3)	19 (21.6)	
White	565 (76.8)	137 (76.5)	60 (68.2)	
Multiple races	10 (1.4)	9 (5.0)	4 (4.5)	
Unknown or not reported	8 (1.1)	5 (2.8)	3 (3.4)	
Sex				
Female	432 (58.7)	80 (44.7)	21 (23.9)	<.001
Male	304 (41.3)	99 (55.3)	67 (76.1)	
Age, mean (SD), y	28.8 (3.7)	28.7 (3.8)	28.3 (3.4)	.44
Income, $				
<50 000	335 (45.5)	94 (52.5)	58 (65.9)	.005
≥50 000 and <100 000	280 (38.0)	56 (31.3)	19 (21.6)	
≥100 000	118 (16.0)	28 (15.6)	10 (11.4)	
Missing	3 (0.4)	1 (0.6)	1 (1.1)	
Education				
High school diploma or GED	126 (17.1)	49 (27.4)	22 (25.0)	<.001
Less than high school diploma	17 (2.3)	5 (2.8)	10 (11.4)	
Some college	129 (17.5)	36 (20.1)	20 (22.7)	
Bachelor’s degree	336 (45.7)	68 (38.0)	29 (33.0)	
Postgraduate degree	128 (17.4)	21 (11.7)	7 (8.0)	
Cannabis dependence^b^	0	40 (22.3)	53 (60.2)	<.001
THC positive test result	19 (2.6)	37 (20.7)	50 (56.8)	<.001
Age at first cannabis use, y				
Never	458 (62.2)	0	0	<.001
<14	11 (1.5)	17 (9.5)	31 (35.2)	
15-17	77 (10.5)	86 (48.0)	33 (37.5)	
18-20	106 (14.4)	58 (32.4)	17 (19.3)	
≥21	84 (11.4)	18 (10.1)	7 (8.0)	
Alcohol dependence^b^	29 (3.9)	20 (11.2)	11 (12.5)	<.001
Nicotine dependence^c^				
Median score (range)	1.00 (0-6.00)	1.00 (0-6.00)	2.50 (0-6.00)	.02
Nonsmoker	627 (85.2)	93 (52.0)	26 (29.5)	
Alcohol *z* score, mean (SD)^d^	−0.05 (0.53)	0.17 (0.39)	0.23 (0.46)	<.001

Abbreviations: GED, general educational development; THC, tetrahydrocannabinol.

^a^ Unless specified otherwise, values are presented as No. (%) of participants.

^b^ Assessed using the Semi-Structured Assessment for the Genetics of Alcoholism using *Diagnostic and Statistical Manual of Mental Disorders, Fourth Edition* criteria.

^c^ Score on the Fagerström Test for Nicotine Dependence.

^d^ Composite score representing frequency and quantity of alcohol consumption.

For the primary analysis, we used 7 linear mixed-effects regression models (ie, 1 model for each of the 7 tasks). For each model, we used the lme4 package in R, version 4.4.0 (R Project for Statistical Computing), to assess associations of the tasks with (1) lifetime history of cannabis (linear and quadratic fit), (2) recent use, and (3) history of dependence. Lifetime history was coded as an ordered factor with 3 levels. Models were adjusted for effects of alcohol, nicotine, race, education, income, sex, and age at first cannabis use (reference: never used). Sibling status was assessed, and this nonindependence was accounted for by a random intercept of a single variable, MotherID, which was represented as a unique value for every biological family and coded as a factor. Given the 7 models, we performed false discovery rate correction for *P* values using the Benjamini-Hochberg method.

For graphical comparison, we calculated effect sizes (Cohen *d*) for activation during each task by (1) lifetime history of use (heavy users vs nonusers), (2) recent use (THC-positive vs THC-negative result), and (3) cannabis diagnosis (history vs no history of dependence). We report comparisons between nonusers and moderate users, as well as heavy users and moderate users, in eFigures 2 and 3 in Supplement 1.

#### Post Hoc and Sensitivity Analyses

For statistically significant results of brain activation, we conducted post hoc analysis to determine which regions were associated with the result. This involved running separate linear mixed-effects models as specified earlier for each brain region (eg, for the working memory task, the 4 regions included the anterior insula [AVI], the superior parietal lobule [7Pm], the medial PFC [8BM], and the dorsolateral PFC [i6-8]).

To examine how brain activation during the tasks are associated with cognitive performance, we fit the linear mixed-effects models described earlier for (1) accuracy during the working memory task, (2) performance on episodic verbal memory, and (3) accuracy during the theory of mind task. To confirm that outcomes associated with lifetime history of heavy use were not associated with recent cannabis use, we excluded all individuals who had a positive test result for THC and we reran the models for the working memory and theory of mind tasks. We assessed the assumption of no unmeasured confounders using the sensemakr package in R, whereby we fit a linear model as specified for our primary analyses and indicated that recent THC use was our benchmark covariate for confounding. This analysis indicates the level that an unmeasured confound would have to be associated with the primary independent variable (history of use) and outcome (activation) to reduce the estimate of the association to 0. We also examined correlation using Spearman rank-ordered correlation for activation during each of the 7 tasks, along with episodic verbal memory, crystallized intelligence, income, and education level (as an ordered factor). We conducted THC-by-sex interaction models for tasks that showed an association with recent use.

## Results

### Participant Characteristics

This study comprised 1003 adults (mean [SD] age, 28.7 [3.7] years), including 470 men (46.9%) and 533 women (53.1%). A total of 2 participants (0.1%) were American Indian or Alaska Native, 63 were Asian (6.3%), 137 were Black (13.7%), 762 were White (76.0%), and 23 (2.3%) were of multiple races; race was unknown or not reported for 16 participants (1.6%). Heavy lifetime users were more likely to be male, to have lower income, and to have lower levels of education than nonusers (χ^2^_2_ = 41.7; *P* < .001) (Table). Heavy lifetime users were also more likely to have positive THC urine screens and a diagnosis of dependence relative to moderate users and nonusers (χ^2^_2_ = 268.2; *P* < .001). Heavy lifetime users also had higher scores for nicotine dependence severity and alcohol use relative to those with negative THC test results as determined via the Wilcoxon rank sum test (*W* > 57 539; *P* < .001).

### Brain Activation Outcomes of Cannabis Use

**Figure 2.**
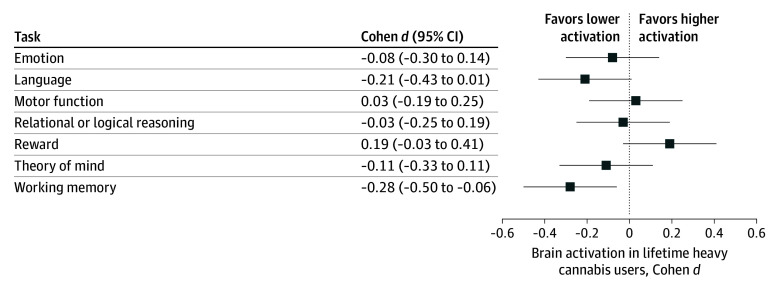
Lifetime History of Cannabis Use and Mean Activation During Each of the 7 Tasks Effect sizes (Cohen *d*) were estimated by comparing lifetime heavy users (n = 88) to nonusers (n = 736). The effect size of lifetime history was statistically significant for the working memory task in the full model that adjusted for recent use and covariates (*P* = .02). The effect size for the theory of mind task showed *P* = .04 in the model but did not survive correction for multiple comparisons.

Although there were no linear associations between lifetime history of cannabis use and task activation after adjusting for associations with recent use, there were significant quadratic effects (β = −4.54 [95% CI, −7.55 to −1.54]; *P* = .003 and Benjamini-Hochberg adjusted *P* = .02), such that greater use was associated with lower activation (full statistical models are shown in eTables 1-7 in Supplement 1). The association between lifetime history of use and theory of mind task activation did not survive correction for multiple comparisons. A summary of effect sizes for brain activation among lifetime heavy users vs nonusers is illustrated in Figure 2; the largest effect size (Cohen *d* = −0.28 [95% CI, −0.50 to −0.06]; false discovery rate corrected *P* = .02) was observed for the working memory task. Central tendency by group is reported for each task in eTable 8 in Supplement 1.

**Figure 3.**
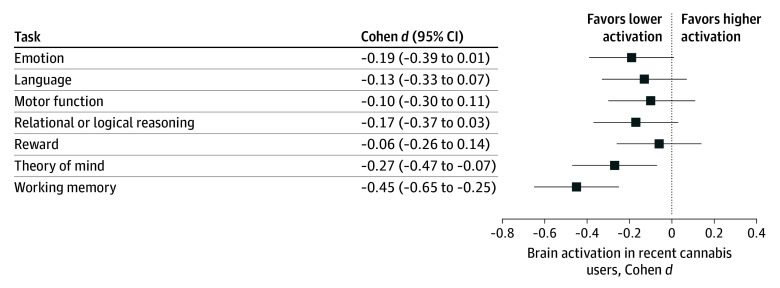
Recent Cannabis Use and Mean Activation During Each of the 7 Tasks The effect size was estimated by comparing individuals with a positive (n = 106) vs negative (n = 899) urine drug screen. Despite the moderate effect sizes observed for the theory of mind task and the working memory task, these effect sizes did not remain statistically significant when adjusting for race and education and when correcting for multiple comparisons.

The comparison of individuals with positive vs negative THC test results revealed lower brain activation in recent cannabis users for the working memory and the theory of mind tasks. However, in the full model, the associations for the theory of mind task no longer remained after adjusting for race and education, because both of these variables were associated with a positive THC test result and also with task activation (eTables 10 and 11 in Supplement 1). After adjusting for associations with lifetime use, the working memory and motor tasks showed associations between recent cannabis use and activation levels but these did not survive false discovery rate correction. A summary of effect sizes for brain activation and positive vs negative urine samples is illustrated in Figure 3, and central tendency by group is reported in eTable 9 in Supplement 1. No task showed an association with diagnosis of cannabis dependence (eFigure 1 and eTable 12 in Supplement 1).

### Post Hoc and Sensitivity Analyses

**Figure 4.**
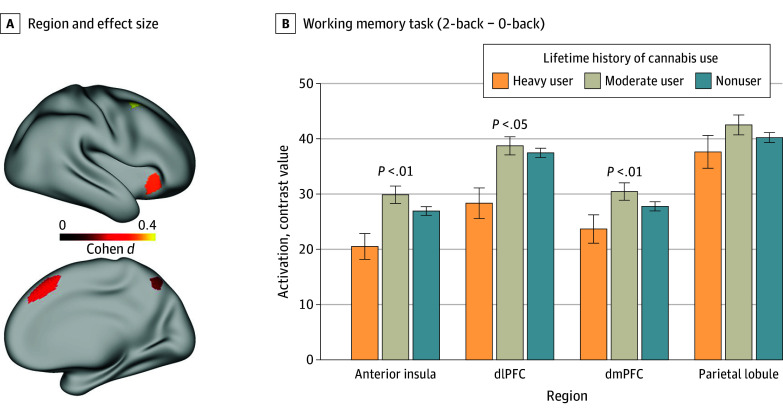
Lifetime History of Cannabis Use and Activation During the Working Memory Task A, Brain images depicting regions and effect size. Each of the 4 regions comprised in the working memory task summary was examined separately as a post hoc analysis to determine which regions were associated with cannabis history. The brain image depicts the effect size of the comparison between heavy and nonusers for each of the 4 regions. B, Bar graph of the models. The models included lifetime history as an independent variable and adjusted for recent cannabis use (ie, positive urine screen), age, sex, education, income, alcohol use, and nicotine use. The graph indicates the mean value by group, and the error bar represents the SEM. *P* values refer to the significance of the quadratic effect of lifetime history of use in the full model, not to post hoc comparisons. dlPFC indicates dorsolateral prefrontal cortex; dmPFC, dorsomedial prefrontal cortex.

For the working memory task, the models for the anterior insula, medial PFC, and dorsolateral PFC showed associations with lifetime history of cannabis use (all *t* < −2.0; all *P* < .02), as shown in Figure 4 (full models are displayed in eTables 13-16 in Supplement 1). However, the model for the superior parietal lobule (*t* = −1.6; *P* = .10) did not.

When removing all individuals with a positive urine screen for THC, 38 individuals remained who had a lifetime history of heavy cannabis use, and the association with lifetime history of heavy use remained for working memory task brain activation (β = −3.66 [95% CI, −7.35 to 0.02]; *P* = .05). The analysis of no unmeasured confounders indicated that a variable could have 3 times the magnitude of association with history of use and brain activation, relative to recent use, and still not reduce the estimate of the primary association to 0, which seems unlikely.

For behavioral performance, recent cannabis use was associated with poorer performance on the working memory task, the episodic verbal memory task, and the theory of mind task (eTables 17-19 in Supplement 1). Lifetime history of heavy use was not associated with performance on these tasks.

Brain activation levels during the relational, theory of mind, and working memory tasks were correlated with crystallized intelligence, education, and scores on the verbal episodic memory task (ρ > 0.13; *P* < .001) (eFigure 4 in Supplement 1). There was no sex-by-THC interaction for working memory but there was for the motor task (*t* = −3.3; *P* = .001), such that women showed no association with THC (*t* = 1.88; *P* = .24) but men showed lower activation levels if they had a positive THC result (*t* = 3.17; *P* = .01) (eFigure 5 in Supplement 1).

## Discussion

In this study, we characterized the association of recent cannabis use and heavy lifetime cannabis use with brain activation across 7 tasks. Heavy lifetime cannabis use was associated with lower brain activation only during the working memory task, and this association remained after excluding individuals with recent use. Brain activation levels during the working memory task were associated with verbal episodic memory performance, intelligence, and education, suggesting that they are meaningful indicators of cognitive function. Although recent cannabis use was associated with lower brain activation during the working memory and motor tasks, the association did not remain after false discovery rate correction. We observed an absence of an association of a dependence diagnosis with brain function, suggesting that factors associated with diagnosis, such as social and legal consequences, may be unrelated to brain function outcomes attributable to cannabis use. Thus, the diagnoses of dependence may be less relevant than recent or cumulative exposure to pharmacologically active components of cannabis (eg, THC).

In this study, lower brain activation during the working memory task in heavy cannabis users was most pronounced in the dorsolateral PFC, dorsomedial PFC, and anterior insula. These are regions that have a relatively high density of CB1 receptors and where receptor availability was found to be reduced in association with daily cannabis exposure.^47^ Similarly, rodent studies showed that THC exposure reduced the density and sensitivity of CB1 receptors in these brain regions,^48^ providing evidence that heavy cannabis use can cause neural adaption. Because THC can reduce CB1 density, this could provide a mechanism to explain findings that cannabis use is associated with lower cortical thickness in the dorsomedial PFC and dorsolateral PFC.^16^ The impact of these putative effects was observed on the working memory task in the current study. A previous study that examined the HCP data also showed that recent cannabis use was associated with lower activation during the working memory task in the anterior insula and middle frontal gyrus, and that their decreased activation mediated the association between cannabis use and poorer performance on an episodic memory task.^49,50^ Our results are consistent with these findings, although they suggest that heavy lifetime cannabis use among participants was associated with lower activation to a working memory task even after removing individuals with a positive urine screen at the time of testing to control for recent use. This finding also accords with evidence that heavy cannabis use alters brain activation in the absence of recent use^51^ and that acute THC administration reduces brain activation in brain regions involved in working memory.^52^

The association we observed between recent use and working memory task activation and performance suggests that abstaining from cannabis prior to cognitively demanding situations will likely help with performance. The exact duration of this period of abstinence is unclear, but studies suggest that residual cognitive effects of cannabis may remain for 2 to 4 weeks after abstinence.^53,54^ Furthermore, in heavy users, abstaining from cannabis may also lead to withdrawal symptoms, which may last for a week or more following cessation and could also affect performance.^55^ Our findings highlight the need to educate cannabis users about the consequences of recent and heavy lifetime cannabis use on cognitively demanding working memory tasks. Similarly, the association between heavy use and decreased brain function could motivate regular cannabis users to reduce their cannabis use and could encourage treatment. Further studies are required to determine guidance on the length of abstinence that may be necessary to improve cognitive performance.

We observed that recent cannabis use was associated with decreased behavioral accuracy in the theory of mind task with similar, albeit not statistically significant, brain activation outcomes for recent use and history of heavy cannabis use. Reduced brain activation to a theory of mind task was reported previously in cannabis users relative to healthy adults, and the study’s authors hypothesized that this could contribute to the increased risk of schizophrenia, a condition associated with profound deficits in theory of mind processes.^56^ Despite this evidence, few studies have investigated theory of mind–related activation in cannabis-using samples; to our knowledge, our study represents a relatively novel contribution. The deficits in theory of mind–related processing and working memory processing may suggest that THC exposure may affect overlapping neural mechanisms that could contribute to observed associations between THC and psychopathology. In our study, we also observed reduced activation in recent cannabis users, which could contribute to the emergence of acute psychoses observed during THC intoxication, particularly for high THC doses.^57^ For the motor task, we observed a significant interaction of sex with brain activation, such that men showed lower activation when they had a positive THC result but women showed no effect of THC. A 2022 review identified 18 studies that examined a sex-by-THC interaction effect^58^; although the majority of these studies showed no interaction, the few that did indicated that women experienced greater effects of cannabis than men.^58^ These effects included smaller orbitofrontal cortex and cerebellar^59^ volumes in women vs men with cannabis dependence.^60^ In addition, relative to nonusers, female (but not male) heavy cannabis users showed a blunted neural response to a stimulant challenge.^61^ Studies specifically designed and powered to assess the interaction of cannabis with sex throughout the lifespan are needed.

### Limitations

This study has limitations. This was an uncontrolled, cross-sectional study, so the observed associations of cannabis with brain function outcomes should not be considered causal. Participants were young adults, so these findings may not generalize to other age groups. History of heavy cannabis use was defined as a lifetime history of greater than 1000 uses or a diagnosis of cannabis dependence, but the sample was recruited from the community, so it may represent a relatively low level of addiction severity. We lacked data to determine when the most recent use occurred or to quantify THC metabolite concentration. It is possible that the association of recent use with brain activation would have been larger in a study where use was determined to be closer to the scan time so that participants experience peak effects of THC during tasks (ie, 0.5-4 hours after use, depending on route of administration). The timing of heavy THC exposure is unknown; although age at first use was not statistically significant in our models, first use is a crude measure, and the timing of heavy use may still matter.^16^ We also lacked data on typical THC dose, potency, additional cannabis constituents (eg, cannabidiol), and route of cannabis administration. Finally, although the sample size was relatively large, some subgroups (eg, women with a positive urine sample) were small, limiting statistical power. Similarly, we could not examine other substance use (eg, opioids) due to low frequency, and we did not examine psychiatric comorbidities.

## Conclusions

In this cross-sectional study of young adults, lifetime heavy cannabis use history was associated with lower brain activation related to working memory, with a small to medium effect size. Before adjustment for covariates and correction for multiple comparisons, recent and lifetime cannabis use were associated with poorer behavioral performance on the theory of mind task; therefore, theory of mind should be examined in future studies. Evidence supported that both recent and heavy lifetime cannabis use were associated with diminished brain activation and cognitive performance during working memory. These findings suggest that large, longitudinal studies are needed to assess the causality of cannabis use toward altering brain function and the duration over which these effects persist.

## Notes

Supplement 1.
eMethods
**eTable 1.** Full Model Examining the Effect of Cannabis Use (History, Recent Use) and Dependence Diagnosis on Activation During the Working Memory Task **eTable 2.** Full Model Examining the Effect of Cannabis Use (History, Recent Use) and Dependence Diagnosis on Activation During the Theory of Mind Task **eTable 3.** Full Model Examining the Effect of Cannabis Use (History, Recent Use) and Dependence Diagnosis on Activation During the Language Task **eTable 4.** Full Model Examining the Effect of Cannabis Use (History, Recent Use) and Dependence Diagnosis on Activation During the Reward Task **eTable 5.** Full Model Examining the Effect of Cannabis Use (History, Recent Use) and Dependence Diagnosis on Activation During the Emotion Task **eTable 6.** Full Model Examining the Effect of Cannabis Use (History, Recent Use) and Dependence Diagnosis on Activation During the Relational Task **eTable 7.** Full Model Examining the Effect of Cannabis Use (History, Recent Use) and Dependence Diagnosis on Activation During the Motor Task **eTable 8.** Mean (SD) Brain Activation Level for Each Task by Lifetime History of Cannabis Use **eTable 9.** Mean (SD) Brain Activation Level for Each Task by Recent Cannabis Use (Tetrahydrocannabinol [THC] Negative vs THC Positive) **eTable 10.** Tetrahydrocannabinol (THC) Status in Urine and Association With Education, Race, and Theory of Mind (TOM) Activation During Functional Magnetic Resonance Imaging **eTable 11.** Association of Education With Theory of Mind (TOM) Activation During Functional Magnetic Resonance Imaging **eTable 12.** Mean (SD) Brain Activation Level for Each Task by Lifetime History of Cannabis Dependence Diagnosis (No vs Yes) **eTable 13.** Full Model Examining the Effect of Cannabis Use (History, Recent Use) and Dependence Diagnosis on Activation of the Right Anterior Ventral Insula During the Working Memory Task **eTable 14.** Full Model Examining the Effect of Cannabis Use (History, Recent Use) and Dependence Diagnosis on Activation of the Right Parietal Lobule [7Pm] During the Working Memory Task **eTable 15.** Full Model Examining the Effect of Cannabis Use (History, Recent Use) and Dependence Diagnosis on Activation of the Dorsomedial Prefrontal Cortex (8BM) During the Working Memory Task **eTable 16.** Full Model Examining the Effect of Cannabis Use (History, Recent Use) and Dependence Diagnosis on Activation of the Dorsolateral Prefrontal Cortex (i6-8) During the Working Memory Task **eTable 17.** Full Model Examining the Effect of Cannabis Use (History, Recent Use) and Dependence Diagnosis on Behavioral Accuracy During the Working Memory Task **eTable 18.** Full Model Examining the Effect of Cannabis Use (History, Recent Use) and Dependence Diagnosis on Behavioral Accuracy During the Episodic Verbal Learning/Memory Task (Penn Word Task), Indexed as the Total Number of Accurate Response Out of 40 **eTable 19.** Full Model Examining the Effect of Cannabis Use (History, Recent Use) and Dependence Diagnosis on Behavioral Accuracy During the Theory of Mind Task, Indexed by the Percentage of Correct Identifications of Social Interactions **eFigure 1.** Effects of Diagnosis of Cannabis Dependence on Average Activation During Each of the 7 Tasks **eFigure 2.** Effects of Lifetime History of Cannabis Use on Average Activation During Each of the 7 Tasks **eFigure 3.** Effects of Lifetime History of Cannabis Use on Average Activation During Each of the 7 Tasks **eFigure 4.** Correlation Matrix Showing the Relationship Between Verbal Episodic Memory and Crystallized Intelligence With Income, Education, and the Average Activation From the 7 Tasks **eFigure 5.** Sex-by-Tetrahydrocannabinol (THC) Interaction During the Motor Task
eReferences

Supplement 2.
Data Sharing Statement
