# Supplementary material for: Brain Function Outcomes of Recent and Lifetime Cannabis Use
Source: JAMA Netw Open. 2025 Jan 28;8(1):e2457069. doi: 10.1001/jamanetworkopen.2024.57069 (PMC11775743; doi:10.1001/jamanetworkopen.2024.57069)
Supplement: Supplement 1. — eMethods eTable 1. Full Model Examining the Effect of Cannabis Use (History, Recent Use) and Dependence Diagnosis on Activation During the Working Memory Task eTable 2. Full Model Examining the Effect of Cannabis Use (History, Recent Use) and Dependence Diagnosis on Activation During the Theory of Mind Task eTable 3. Full Model Examining the Effect of Cannabis Use (History, Recent Use) and Dependence Diagnosis on Activation During the Language Task eTable 4. Full Model Examining the Effect of Cannabis Use (History, Recent Use) and Dependence Diagnosis on Activation During the Reward Task eTable 5. Full Model Examining the Effect of Cannabis Use (History, Recent Use) and Dependence Diagnosis on Activation During the Emotion Task eTable 6. Full Model Examining the Effect of Cannabis Use (History, Recent Use) and Dependence Diagnosis on Activation During the Relational Task eTable 7. Full Model Examining the Effect of Cannabis Use (History, Recent Use) and Dependence Diagnosis on Activation During the Motor Task eTable 8. Mean (SD) Brain Activation Level for Each Task by Lifetime History of Cannabis Use eTable 9. Mean (SD) Brain Activation Level for Each Task by Recent Cannabis Use (Tetrahydrocannabinol [THC] Negative vs THC Positive) eTable 10. Tetrahydrocannabinol (THC) Status in Urine and Association With Education, Race, and Theory of Mind (TOM) Activation During Functional Magnetic Resonance Imaging eTable 11. Association of Education With Theory of Mind (TOM) Activation During Functional Magnetic Resonance Imaging eTable 12. Mean (SD) Brain Activation Level for Each Task by Lifetime History of Cannabis Dependence Diagnosis (No vs Yes) eTable 13. Full Model Examining the Effect of Cannabis Use (History, Recent Use) and Dependence Diagnosis on Activation of the Right Anterior Ventral Insula During the Working Memory Task eTable 14. Full Model Examining the Effect of Cannabis Use (History, Recent Use) and Dependence Diagnosis on Activation of the Right Parietal Lobu [file jamanetwopen-e2457069-s001.pdf]

Supplementary Online Content

Gowin JL, Ellingson JM, Karoly HK, et al. Brain function outcomes of recent and lifetime cannabis use. *JAMA Netw Open*. 2025;8(1):e2457069.
doi:10.1001/jamanetworkopen.2024.57069

eMethods

eTable 1. Full Model Examining the Effect of Cannabis Use (History, Recent Use) and Dependence Diagnosis on Activation During the Working Memory Task

eTable 2. Full Model Examining the Effect of Cannabis Use (History, Recent Use) and Dependence Diagnosis on Activation During the Theory of Mind Task

eTable 3. Full Model Examining the Effect of Cannabis Use (History, Recent Use) and Dependence Diagnosis on Activation During the Language Task

eTable 4. Full Model Examining the Effect of Cannabis Use (History, Recent Use) and Dependence Diagnosis on Activation During the Reward Task

eTable 5. Full Model Examining the Effect of Cannabis Use (History, Recent Use) and Dependence Diagnosis on Activation During the Emotion Task

eTable 6. Full Model Examining the Effect of Cannabis Use (History, Recent Use) and Dependence Diagnosis on Activation During the Relational Task

eTable 7. Full Model Examining the Effect of Cannabis Use (History, Recent Use) and Dependence Diagnosis on Activation During the Motor Task

eTable 8. Mean (SD) Brain Activation Level for Each Task by Lifetime History of Cannabis Use

eTable 9. Mean (SD) Brain Activation Level for Each Task by Recent Cannabis Use (Tetrahydrocannabinol [THC] Negative vs THC Positive)

eTable 10. Tetrahydrocannabinol (THC) Status in Urine and Association With Education, Race, and Theory of Mind (TOM) Activation During Functional Magnetic Resonance Imaging

eTable 11. Association of Education With Theory of Mind (TOM) Activation During Functional Magnetic Resonance Imaging

eTable 12. Mean (SD) Brain Activation Level for Each Task by Lifetime History of Cannabis Dependence Diagnosis (No vs Yes)

eTable 13. Full Model Examining the Effect of Cannabis Use (History, Recent Use) and Dependence Diagnosis on Activation of the Right Anterior Ventral Insula During the Working Memory Task

© 2025 Gowin JL et al. *JAMA Network Open.*

eTable 14. Full Model Examining the Effect of Cannabis Use (History, Recent Use) and Dependence Diagnosis on Activation of the Right Parietal Lobule [7Pm] During the Working Memory Task

eTable 15. Full Model Examining the Effect of Cannabis Use (History, Recent Use) and Dependence Diagnosis on Activation of the Dorsomedial Prefrontal Cortex (8BM) During the Working Memory Task

eTable 16. Full Model Examining the Effect of Cannabis Use (History, Recent Use) and Dependence Diagnosis on Activation of the Dorsolateral Prefrontal Cortex (i6-8) During the Working Memory Task

eTable 17. Full Model Examining the Effect of Cannabis Use (History, Recent Use) and Dependence Diagnosis on Behavioral Accuracy During the Working Memory Task

eTable 18. Full Model Examining the Effect of Cannabis Use (History, Recent Use) and Dependence Diagnosis on Behavioral Accuracy During the Episodic Verbal Learning/Memory Task (Penn Word Task), Indexed as the Total Number of Accurate Response Out of 40

eTable 19. Full Model Examining the Effect of Cannabis Use (History, Recent Use) and Dependence Diagnosis on Behavioral Accuracy During the Theory of Mind Task, Indexed by the Percentage of Correct Identifications of Social Interactions

eFigure 1. Effects of Diagnosis of Cannabis Dependence on Average Activation During Each of the 7 Tasks

eFigure 2. Effects of Lifetime History of Cannabis Use on Average Activation During Each of the 7 Tasks

eFigure 3. Effects of Lifetime History of Cannabis Use on Average Activation During Each of the 7 Tasks

eFigure 4. Correlation Matrix Showing the Relationship Between Verbal Episodic Memory and Crystallized Intelligence With Income, Education, and the Average Activation From the 7 Tasks

eFigure 5. Sex-by-Tetrahydrocannabinol (THC) Interaction During the Motor Task

eReferences

This supplementary material has been provided by the authors to give readers additional information about their work.

© 2025 Gowin JL et al. *JAMA Network Open.*

eMethods

Brain regions associated with activation during primary contrasts for each task have been meticulously described in the multi-modal parcellation of the cortex, based on the HCP data[1]. For each task, we list the regions activated by the contrast, based on the text description and the figures from the primary analysis of the data[1]; the primary analysis used a maximum $p<0.001$ for significance, with most effect sizes of Cohen's $d > 1.00$[1]. We used the mean value of the activation levels across the listed regions for each task, so each participant had a single value representing activation level for each task.

- Emotion Task: Participants alternated between blocks of seeing faces (angry or fearful) or shapes[2]. The contrast of interest was faces > shapes. Regions activated by this contrast included the amygdala, fusiform face complex, temporal parieto-occipital junction, anterior prefrontal cortex, and inferior frontal gyrus.
- Gambling Task: Participants played a card guessing game to win money[3]. They pressed one of two buttons to guess if a mystery card was higher or lower than 5. They won $1 if correct or lost $0.50 if incorrect. No change in earnings occurred if the card was a 5. The contrast of interest was reward > punishment. Regions activated by this contrast included the nucleus accumbens, inferior parietal, and inferior frontal cortex.
- Language Task: The task consisted of alternating blocks of stories and math tasks[4]. During story blocks, participants listened to stories adapted from Aesop's fables, and then answered questions by indicating which of two options was the correct topic of the story. During math blocks, participants performed arithmetic. The contrast of interest was story > math. Regions activated by this contrast included 55b, peri-sylvian language area, superior frontal language area, and Broca's area.
- Motor Task: Participants were instructed to move body parts (e.g., tap finger, squeeze toe)[5,6] to map motor areas of the brain. The contrast of interest was

© 2025 Gowin JL et al. *JAMA Network Open.*

movement > baseline (i.e., periods of rest, fixation). Regions activated by this contrast included supplementary motor area, the primary area, operculum, posterior cingulate, and anterior cingulate cortex.

- Relational Task: This relational matching-to-sample task[7] requires the participants to assess how two pairs of objects are similar[8], like the cognitive skill tested by a Raven's Progressive Matrix task[9]. Participants alternated between two conditions: a simple matching-to-sample (e.g., are the objects both triangles?) or a relational-comparison decisions (e.g., if the top pair of objects differs on pattern fill, does the bottom also differ on pattern fill, such as polka dot?). The contrast of interest was relational > match. Regions activated by this contrast included ventromedial prefrontal cortex, anterior ventral insula, orbitofrontal cortex, inferior frontal gyrus, and dorsolateral prefrontal cortex.
- Theory of Mind Task: Participants saw videos of shapes moving either randomly or in ways that indicated theory-of-mind (i.e., consideration of other's feelings, such as dancing together)[10,11]. The contrast of interest was theory-of-mind > random. Regions activated by this contrast included premotor cortex, superior temporal sulcus, peri-hippocampal area, lateral temporal cortex, temporo-parietal junction, and triangular part of the inferior frontal gyrus.
- Working Memory Task: Participants completed an n-back task which involved a series of images[12,13]. In the 2-back condition, they clicked a button when the current image matched the image they saw two images prior. In the 0-back condition, they clicked a button when they saw the target image. The contrast of interest was 2-back > 0-back. Regions activated by this contrast included anterior ventral insula, superior parietal cortex, and dorsomedial and dorsolateral prefrontal cortex.

© 2025 Gowin JL et al. *JAMA Network Open.*

eTable 1. Full Model Examining the Effect of Cannabis Use (History, Recent Use) and Dependence Diagnosis on Activation During the Working Memory Task

Lifetime history considers number of cannabis uses with levels for 0-10, 11-999, and 1000+ entered as ordered factors (levels 0, 1, 2). Linear and quadratic effects are fit. Dependence diagnosis is based on DSM-IV criteria. THC indicates recent use. For race, the reference group is white. For 1st use (of cannabis), the reference group is "Never". The p-values are uncorrected.

	**Working Memory**		
Predictors	*Estimates*	*CI*	*p*
(Intercept)	0.78	-11.07 – 12.63	0.898
Lifetime History [linear]	-2.16	-6.61 – 2.29	0.342
Lifetime History [quadratic]	-4.54	-7.55 – -1.54	**0.003**
THC [Positive]	-4.70	-9.35 – -0.05	**0.048**
Dependence Dx [Yes]	2.50	-2.63 – 7.63	0.339
1st Use <14	-0.49	-6.82 – 5.85	0.880
1st Use 15-17	-1.13	-5.05 – 2.78	0.570
1st Use 18-20	-1.05	-4.63 – 2.52	0.564
1st Use 21+	-2.27	-6.32 – 1.78	0.272
Education	2.18	1.42 – 2.94	**<0.001**
Race [Am. Indian/Alaskan Nat.]	3.01	-22.72 – 28.73	0.819
Race [Asian/Nat. Hawaiian/Othr Pacific Is.]	3.07	-2.10 – 8.23	0.244
Race [Black or African Am.]	-6.58	-10.57 – -2.59	**0.001**
Race [More than one]	5.89	-2.33 – 14.11	0.160
Race [Unknown or Not Reported]	-0.59	-10.02 – 8.84	0.902
Sex [M]	3.53	1.05 – 6.01	**0.005**
Income	-0.12	-0.71 – 0.48	0.705
Alcohol Z	0.10	-2.39 – 2.60	0.934
Tobacco Z	-0.56	-2.19 – 1.06	0.497
**Random Effects**			
σ^2	300.68		
$\tau_{00\ \text{Family Structure}}$	47.11		
ICC	0.14		
$N_{\text{Family Structure}}$	434		
Observations	998		
Marginal R^2 / Conditional R^2	0.101 / 0.223		

© 2025 Gowin JL et al. *JAMA Network Open.*

eTable 2. Full Model Examining the Effect of Cannabis Use (History, Recent Use) and Dependence Diagnosis on Activation During the Theory of Mind Task

Lifetime history considers number of cannabis uses with levels for 0-10, 11-999, and 1000+ entered as ordered factors (levels 0, 1, 2). Linear and quadratic effects are fit. Dependence diagnosis is based on DSM-IV criteria. THC indicates recent use. For race, the reference group is white. For 1st use (of cannabis), the reference group is “Never”. The p-values are uncorrected.

	**Theory of Mind**		
Predictors	*Estimates*	*CI*	*p*
(Intercept)	20.97	11.90 – 30.05	**<0.001**
Lifetime History [linear]	0.66	-2.79 – 4.11	0.709
Lifetime History [quadratic]	-2.44	-4.77 – -0.11	**0.041**
THC [Positive]	-2.63	-6.23 – 0.97	0.152
Dependence Dx [Yes]	1.10	-2.90 – 5.09	0.591
1st Use <14	-0.14	-4.99 – 4.71	0.954
1st Use 15-17	-0.91	-3.94 – 2.11	0.554
1st Use 18-20	-0.31	-3.08 – 2.46	0.826
1st Use 21+	-2.71	-5.86 – 0.43	0.091
Education	0.65	0.07 – 1.23	**0.029**
Race [Am. Indian/Alaskan Nat.]	13.01	-7.16 – 33.18	0.206
Race [Asian/Nat. Hawaiian/Othr Pacific Is.]	4.37	0.53 – 8.20	**0.026**
Race [Black or African Am.]	-4.45	-7.38 – -1.52	**0.003**
Race [More than one]	-1.13	-7.39 – 5.13	0.722
Race [Unknown or Not Reported]	-0.65	-7.91 – 6.62	0.862
Sex [M]	0.21	-1.69 – 2.11	0.827
Income	0.02	-0.44 – 0.48	0.930
Alcohol Z	0.78	-1.13 – 2.70	0.422
Tobacco Z	-0.75	-2.00 – 0.50	0.240
**Random Effects**			
σ^2	198.20		
$\tau_{00\ \text{Family Structure}}$	8.93		
ICC	0.04		
$N_{\text{Family Structure}}$	434		
Observations	998		
Marginal R^2 / Conditional R^2	0.047 / 0.088		

© 2025 Gowin JL et al. *JAMA Network Open.*

eTable 3. Full Model Examining the Effect of Cannabis Use (History, Recent Use) and Dependence Diagnosis on Activation During the Language Task

Lifetime history considers number of cannabis uses with levels for 0-10, 11-999, and 1000+ entered as ordered factors (levels 0, 1, 2). Linear and quadratic effects are fit. Dependence diagnosis is based on DSM-IV criteria. THC indicates recent use. For race, the reference group is white. For 1st use (of cannabis), the reference group is "Never". The p-values are uncorrected.

	**Language**		
Predictors	*Estimates*	*CI*	*p*
(Intercept)	-11.61	-23.10 – -0.12	**0.048**
Lifetime History [linear]	-2.49	-6.74 – 1.77	0.251
Lifetime History [quadratic]	-1.36	-4.22 – 1.51	0.353
THC [Positive]	0.88	-3.58 – 5.33	0.700
Dependence Dx [Yes]	2.34	-2.52 – 7.20	0.345
1st Use <14	1.70	-4.43 – 7.83	0.587
1st Use 15-17	0.41	-3.33 – 4.16	0.829
1st Use 18-20	-0.63	-4.05 – 2.78	0.716
1st Use 21+	0.41	-3.45 – 4.28	0.834
Education	0.42	-0.32 – 1.15	0.263
Race [Am. Indian/Alaskan Nat.]	-17.79	-41.91 – 6.33	0.148
Race [Asian/Nat. Hawaiian/Othr Pacific Is.]	5.33	0.09 – 10.58	**0.046**
Race [Black or African Am.]	-2.63	-6.72 – 1.47	0.208
Race [More than one]	1.25	-6.78 – 9.28	0.760
Race [Unknown or Not Reported]	0.42	-8.67 – 9.51	0.928
Sex [M]	2.94	0.53 – 5.34	**0.017**
Income	0.80	0.23 – 1.37	**0.006**
Alcohol Z	-0.20	-2.62 – 2.21	0.869
Tobacco Z	-0.64	-2.20 – 0.92	0.422
**Random Effects**			
σ^2	247.10		
τ_{00} Family Structure	84.70		
ICC	0.26		
N Family Structure	434		
Observations	998		
Marginal R^2 / Conditional R^2	0.036 / 0.282		

© 2025 Gowin JL et al. *JAMA Network Open.*

eTable 4. Full Model Examining the Effect of Cannabis Use (History, Recent Use) and Dependence Diagnosis on Activation During the Reward Task

Lifetime history considers number of cannabis uses with levels for 0-10, 11-999, and 1000+ entered as ordered factors (levels 0, 1, 2). Linear and quadratic effects are fit. Dependence diagnosis is based on DSM-IV criteria. THC indicates recent use. For race, the reference group is white. For 1st use (of cannabis), the reference group is "Never". The p-values are uncorrected.

	**Reward**		
Predictors	*Estimates*	*CI*	*p*
(Intercept)	13.54	5.79 – 21.28	**0.001**
Lifetime History [linear]	1.80	-1.15 – 4.75	0.231
Lifetime History [quadratic]	0.84	-1.16 – 2.83	0.410
THC [Positive]	-2.33	-5.40 – 0.74	0.137
Dependence Dx [Yes]	0.49	-2.92 – 3.90	0.779
1st Use <14	2.47	-1.66 – 6.61	0.241
1st Use 15-17	1.22	-1.37 – 3.80	0.355
1st Use 18-20	-0.40	-2.76 – 1.96	0.740
1st Use 21+	0.03	-2.65 – 2.71	0.983
Education	-0.20	-0.69 – 0.30	0.438
Race [Am. Indian/Alaskan Nat.]	-9.87	-27.09 – 7.35	0.261
Race [Asian/Nat. Hawaiian/Othr Pacific Is.]	1.09	-2.19 – 4.37	0.516
Race [Black or African Am.]	0.55	-1.95 – 3.05	0.667
Race [More than one]	-1.44	-6.78 – 3.91	0.598
Race [Unknown or Not Reported]	-4.07	-10.28 – 2.13	0.198
Sex [M]	-0.70	-2.32 – 0.92	0.395
Income	-0.06	-0.45 – 0.33	0.768
Alcohol Z	0.76	-0.87 – 2.40	0.359
Tobacco Z	-0.30	-1.37 – 0.77	0.584
**Random Effects**			
σ^2	144.25		
τ_{00} Family Structure	6.69		
ICC	0.04		
N Family Structure	434		
Observations	998		
Marginal R^2 / Conditional R^2	0.014 / 0.058		

© 2025 Gowin JL et al. *JAMA Network Open.*

eTable 5. Full Model Examining the Effect of Cannabis Use (History, Recent Use) and Dependence Diagnosis on Activation During the Emotion Task

Lifetime history considers number of cannabis uses with levels for 0-10, 11-999, and 1000+ entered as ordered factors (levels 0, 1, 2). Linear and quadratic effects are fit. Dependence diagnosis is based on DSM-IV criteria. THC indicates recent use. For race, the reference group is white. For 1st use (of cannabis), the reference group is "Never". The p-values are uncorrected.

	**Emotion**		
Predictors	*Estimates*	*CI*	*p*
(Intercept)	44.88	35.17 – 54.59	**<0.001**
Lifetime History [linear]	0.16	-3.47 – 3.80	0.929
Lifetime History [quadratic]	0.42	-2.04 – 2.87	0.740
THC [Positive]	-2.50	-6.30 – 1.31	0.198
Dependence Dx [Yes]	1.43	-2.76 – 5.61	0.504
1st Use <14	2.77	-2.42 – 7.96	0.295
1st Use 15-17	2.49	-0.71 – 5.69	0.126
1st Use 18-20	0.66	-2.26 – 3.58	0.656
1st Use 21+	2.06	-1.25 – 5.37	0.222
Education	0.08	-0.55 – 0.70	0.811
Race [Am. Indian/Alaskan Nat.]	-4.20	-25.14 – 16.74	0.694
Race [Asian/Nat. Hawaiian/Othr Pacific Is.]	2.19	-2.07 – 6.46	0.313
Race [Black or African Am.]	-0.12	-3.42 – 3.18	0.944
Race [More than one]	-1.62	-8.36 – 5.12	0.637
Race [Unknown or Not Reported]	-5.09	-12.81 – 2.63	0.196
Sex [M]	-3.14	-5.18 – -1.11	**0.002**
Income	-0.05	-0.54 – 0.43	0.832
Alcohol Z	0.41	-1.64 – 2.46	0.694
Tobacco Z	-1.02	-2.35 – 0.31	0.134
**Random Effects**			
σ^2	196.45		
τ_{00} Family Structure	37.00		
ICC	0.16		
N Family Structure	434		
Observations	998		
Marginal R^2 / Conditional R^2	0.021 / 0.176		

© 2025 Gowin JL et al. *JAMA Network Open.*

eTable 6. Full Model Examining the Effect of Cannabis Use (History, Recent Use) and Dependence Diagnosis on Activation During the Relational Task

Lifetime history considers number of cannabis uses with levels for 0-10, 11-999, and 1000+ entered as ordered factors (levels 0, 1, 2). Linear and quadratic effects are fit. Dependence diagnosis is based on DSM-IV criteria. THC indicates recent use. For race, the reference group is white. For 1st use (of cannabis), the reference group is "Never". The p-values are uncorrected.

	**Relational**		
Predictors	*Estimates*	*CI*	*p*
(Intercept)	-1.91	-13.88 – 10.07	0.755
Lifetime History [linear]	0.97	-3.54 – 5.47	0.674
Lifetime History [quadratic]	-1.24	-4.28 – 1.80	0.425
THC [Positive]	-0.63	-5.33 – 4.07	0.791
Dependence Dx [Yes]	0.89	-4.29 – 6.08	0.736
1st Use <14	-2.38	-8.78 – 4.02	0.466
1st Use 15-17	2.30	-1.66 – 6.26	0.254
1st Use 18-20	-2.06	-5.67 – 1.56	0.264
1st Use 21+	4.32	0.22 – 8.41	**0.039**
Education	1.40	0.64 – 2.17	**<0.001**
Race [Am. Indian/Alaskan Nat.]	-2.53	-28.55 – 23.49	0.849
Race [Asian/Nat. Hawaiian/Othr Pacific Is.]	-2.62	-7.84 – 2.60	0.325
Race [Black or African Am.]	-7.71	-11.74 – -3.68	**<0.001**
Race [More than one]	-1.23	-9.53 – 7.08	0.772
Race [Unknown or Not Reported]	2.35	-7.19 – 11.88	0.629
Sex [M]	-1.51	-4.02 – 1.00	0.237
Income	-0.20	-0.80 – 0.41	0.525
Alcohol Z	2.97	0.44 – 5.49	**0.021**
Tobacco Z	-0.24	-1.88 – 1.41	0.778
**Random Effects**			
σ^2	308.09		
τ_{00} Family Structure	47.33		
ICC	0.13		
N Family Structure	434		
Observations	998		
Marginal R^2 / Conditional R^2	0.059 / 0.185		

© 2025 Gowin JL et al. *JAMA Network Open.*

eTable 7. Full Model Examining the Effect of Cannabis Use (History, Recent Use) and Dependence Diagnosis on Activation During the Motor Task

Lifetime history considers number of cannabis uses with levels for 0-10, 11-999, and 1000+ entered as ordered factors (levels 0, 1, 2). Linear and quadratic effects are fit. Dependence diagnosis is based on DSM-IV criteria. THC indicates recent use. For race, the reference group is white. For 1st use (of cannabis), the reference group is "Never". The p-values are uncorrected.

	**Motor**		
Predictors	*Estimates*	*CI*	*p*
(Intercept)	-67.96	-85.39 – -50.54	**<0.001**
Lifetime History [linear]	-1.25	-7.84 – 5.33	0.709
Lifetime History [quadratic]	-2.03	-6.48 – 2.41	0.370
THC [Positive]	-7.81	-14.68 – -0.95	**0.026**
Dependence Dx [Yes]	4.94	-2.66 – 12.53	0.203
1st Use <14	-0.10	-9.41 – 9.22	0.983
1st Use 15-17	-2.99	-8.77 – 2.79	0.311
1st Use 18-20	-1.04	-6.32 – 4.24	0.699
1st Use 21+	-0.00	-5.99 – 5.99	1.000
Education	0.03	-1.08 – 1.15	0.952
Race [Am. Indian/Alaskan Nat.]	-6.28	-44.51 – 31.95	0.747
Race [Asian/Nat. Hawaiian/Othr Pacific Is.]	2.14	-5.36 – 9.64	0.575
Race [Black or African Am.]	2.71	-3.05 – 8.48	0.356
Race [More than one]	2.23	-9.83 – 14.29	0.717
Race [Unknown or Not Reported]	9.60	-4.30 – 23.51	0.176
Sex [M]	7.00	3.35 – 10.64	**<0.001**
Income	-0.36	-1.24 – 0.52	0.422
Alcohol Z	-1.89	-5.57 – 1.79	0.313
Tobacco Z	2.57	0.18 – 4.97	**0.035**
**Random Effects**			
σ^2	682.61		
τ_{00} Family Structure	72.50		
ICC	0.10		
N Family Structure	434		
Observations	998		
Marginal R^2 / Conditional R^2	0.029 / 0.122		

© 2025 Gowin JL et al. *JAMA Network Open.*

eTable 8. Mean (SD) Brain Activation Level for Each Task by Lifetime History of Cannabis Use

	Non-User (N=736)	Moderate User (N=179)	Heavy User (N=88)
**Language**	1.8 (18.1)	2.0 (18.3)	-2.1 (21.3)
**Working Memory**	33.1 (19.5)	35.4 (18.7)	27.5 (20.4)
**Motor**	-66.6 (27.3)	-64.6 (27.3)	-65.8 (31.7)
**Emotion**	45.3 (15.4)	45.1 (15.9)	44.1 (13.7)
**Reward**	9.2 (12.1)	9.5 (12.9)	11.5 (12.1)
**Relational**	15.6 (19.4)	17.8 (18.7)	15.0 (19.2)
**TOM**	28.9 (14.7)	31.0 (13.8)	27.2 (15.3)

eTable 9. Mean (SD) Brain Activation Level for Each Task by Recent Cannabis Use (Tetrahydrocannabinol [THC] Negative vs THC Positive)

	Negative (N=899)	Positive (N=106)
**Language**	1.8 (18.4)	-0.6 (18.9)
**Working Memory**	33.9 (19.1)	25.1 (21.7)
**Motor**	-66.0 (27.0)	-68.6 (32.6)
**Emotion**	45.5 (15.1)	42.6 (16.7)
**Reward**	9.6 (12.0)	8.9 (14.3)
**Relational**	16.2 (19.1)	12.9 (21.4)
**TOM**	29.6 (14.3)	25.6 (16.9)

© 2025 Gowin JL et al. *JAMA Network Open.*

eTable 10. Tetrahydrocannabinol (THC) Status in Urine and Association With Education, Race, and Theory of Mind (TOM) Activation During Functional Magnetic Resonance Imaging

Theory of Mind, Education and Race by THC

	Negative (N=899)	Positive (N=106)	P-value
**Education**			
HS Diploma/GED	151 (16.8%)	46 (43.4%)	<0.001
< HS Diploma	21 (2.3%)	11 (10.4%)	
Some College	166 (18.5%)	19 (17.9%)	
Bachelor	406 (45.2%)	27 (25.5%)	
Post Graduate Degree	153 (17.0%)	3 (2.8%)	
Missing	2 (0.2%)	0 (0%)	
**Race**			
Am. Indian/Alaskan Nat.	1 (0.1%)	1 (0.9%)	<0.001
Asian/Nat. Hawaiian/Othr Pacific Is.	59 (6.6%)	4 (3.8%)	
Black or African Am.	101 (11.2%)	36 (34.0%)	
More than one	18 (2.0%)	5 (4.7%)	
Unknown or Not Reported	13 (1.4%)	3 (2.8%)	
White	707 (78.6%)	57 (53.8%)	
**TOM**			
Mean (SD)	29.6 (14.3)	25.6 (16.9)	0.01

© 2025 Gowin JL et al. *JAMA Network Open.*

eTable 11. Association of Education With Theory of Mind (TOM) Activation During Functional Magnetic Resonance Imaging

Theory-of-Mind Activation by Education

	< HS Diploma (N=32)	HS Diploma / GED (N=197)	Some College (N=185)	Bachelor (N=433)	Post Graduate Degree (N=156)	P-value
**TOM**						
Mean (SD)	25.5 (18.1)	25.9 (15.5)	29.8 (14.9)	30.2 (14.2)	30.3 (12.8)	0.004

eTable 12. Mean (SD) Brain Activation Level for Each Task by Lifetime History of Cannabis Dependence Diagnosis (No vs Yes)

	No (N=912)	Yes (N=93)
**Language**	1.5 (18.5)	1.6 (17.7)
**Working Memory**	33.0 (19.7)	32.6 (18.5)
**Motor**	-66.7 (27.7)	-61.8 (27.4)
**Emotion**	45.2 (15.3)	45.1 (15.0)
**Reward**	9.3 (12.3)	11.3 (12.0)
**Relational**	15.7 (19.5)	17.2 (18.0)
**TOM**	29.1 (14.7)	29.4 (13.9)

© 2025 Gowin JL et al. *JAMA Network Open.*

eTable 13. Full Model Examining the Effect of Cannabis Use (History, Recent Use) and Dependence Diagnosis on Activation of the Right Anterior Ventral Insula During the Working Memory Task

Lifetime history considers number of cannabis uses with levels for 0-10, 11-999, and 1000+ entered as ordered factors (levels 0, 1, 2). Linear and quadratic effects are fit. Dependence diagnosis is based on DSM-IV criteria. THC indicates recent use. For race, the reference group is white. For 1st use (of cannabis), the reference group is "Never". The p-values are uncorrected.

	**Anterior Ventral Insula during Working Memory**		
Predictors	*Estimates*	*CI*	*p*
(Intercept)	-3.66	-17.12 – 9.80	0.594
Lifetime History [linear]	-2.54	-7.62 – 2.55	0.328
Lifetime History [quadratic]	-5.48	-8.91 – -2.04	**0.002**
THC [Positive]	-5.39	-10.70 – -0.09	**0.046**
Dependence Dx [Yes]	1.62	-4.24 – 7.49	0.587
1st Use <14	0.97	-6.22 – 8.17	0.791
1st Use 15-17	-0.66	-5.13 – 3.81	0.772
1st Use 18-20	-2.05	-6.13 – 2.04	0.326
1st Use 21+	-1.01	-5.64 – 3.62	0.668
Education	2.02	1.15 – 2.88	**<0.001**
Race [Am. Indian/Alaskan Nat.]	1.43	-28.09 – 30.95	0.924
Race [Asian/Nat. Hawaiian/Othr Pacific Is.]	4.34	-1.46 – 10.13	0.142
Race [Black or African Am.]	-4.34	-8.80 – 0.11	0.056
Race [More than one]	-2.30	-11.61 – 7.02	0.629
Race [Unknown or Not Reported]	1.04	-9.70 – 11.78	0.850
Sex [M]	4.13	1.31 – 6.94	**0.004**
Income	-0.07	-0.75 – 0.62	0.851
Alcohol Z	0.77	-2.07 – 3.61	0.593
Tobacco Z	-0.21	-2.06 – 1.64	0.824
**Random Effects**			
σ^2	406.43		
τ_{00} Family Structure	44.01		
ICC	0.10		
N Family Structure	434		
Observations	998		
Marginal R^2 / Conditional R^2	0.071 / 0.162		

© 2025 Gowin JL et al. *JAMA Network Open.*

eTable 14. Full Model Examining the Effect of Cannabis Use (History, Recent Use) and Dependence Diagnosis on Activation of the Right Parietal Lobule [7Pm] During the Working Memory Task

Lifetime history considers number of cannabis uses with levels for 0-10, 11-999, and 1000+ entered as ordered factors (levels 0, 1, 2). Linear and quadratic effects are fit. Dependence diagnosis is based on DSM-IV criteria. THC indicates recent use. For race, the reference group is white. For 1st use (of cannabis), the reference group is "Never". The p-values are uncorrected.

	**Parietal Lobule during Working Memory**		
Predictors	*Estimates*	*CI*	*p*
(Intercept)	1.22	-13.41 – 15.86	0.870
Lifetime History [linear]	0.69	-4.80 – 6.19	0.805
Lifetime History [quadratic]	-3.12	-6.83 – 0.59	0.099
THC [Positive]	-5.42	-11.15 – 0.32	0.064
Dependence Dx [Yes]	2.80	-3.53 – 9.12	0.386
1st Use <14	-1.29	-9.11 – 6.53	0.746
1st Use 15-17	-1.61	-6.45 – 3.22	0.512
1st Use 18-20	-0.61	-5.02 – 3.80	0.786
1st Use 21+	-2.72	-7.72 – 2.28	0.286
Education	2.58	1.64 – 3.52	**<0.001**
Race [Am. Indian/Alaskan Nat.]	4.34	-27.37 – 36.05	0.788
Race [Asian/Nat. Hawaiian/Othr Pacific Is.]	6.43	0.03 – 12.83	**0.049**
Race [Black or African Am.]	-11.27	-16.21 – -6.32	**<0.001**
Race [More than one]	11.39	1.24 – 21.55	**0.028**
Race [Unknown or Not Reported]	-0.69	-12.33 – 10.96	0.908
Sex [M]	6.82	3.75 – 9.88	**<0.001**
Income	0.06	-0.68 – 0.79	0.880
Alcohol Z	-0.77	-3.85 – 2.32	0.625
Tobacco Z	-1.04	-3.05 – 0.97	0.309
**Random Effects**			
σ^2	454.89		
τ_{00} Family Structure	75.65		
ICC	0.14		
N Family Structure	434		
Observations	998		
Marginal R^2 / Conditional R^2	0.126 / 0.251		

© 2025 Gowin JL et al. *JAMA Network Open.*

eTable 15. Full Model Examining the Effect of Cannabis Use (History, Recent Use) and Dependence Diagnosis on Activation of the Dorsomedial Prefrontal Cortex (8BM) During the Working Memory Task

Lifetime history considers number of cannabis uses with levels for 0-10, 11-999, and 1000+ entered as ordered factors (levels 0, 1, 2). Linear and quadratic effects are fit. Dependence diagnosis is based on DSM-IV criteria. THC indicates recent use. For race, the reference group is white. For 1st use (of cannabis), the reference group is “Never”. The p-values are uncorrected.

	**Dorsomedial PFC during Working Memory**		
Predictors	*Estimates*	*CI*	*p*
(Intercept)	3.61	-10.40 – 17.62	0.613
Lifetime History [linear]	-1.84	-7.16 – 3.48	0.497
Lifetime History [quadratic]	-4.85	-8.44 – -1.25	**0.008**
THC [Positive]	-3.16	-8.70 – 2.39	0.264
Dependence Dx [Yes]	3.54	-2.61 – 9.70	0.259
1st Use <14	-0.80	-8.29 – 6.69	0.834
1st Use 15-17	-2.76	-7.43 – 1.91	0.246
1st Use 18-20	-1.53	-5.80 – 2.74	0.481
1st Use 21+	-3.33	-8.18 – 1.51	0.177
Education	1.85	0.95 – 2.75	**<0.001**
Race [Am. Indian/Alaskan Nat.]	5.29	-25.75 – 36.32	0.738
Race [Asian/Nat. Hawaiian/Othr Pacific Is.]	-0.01	-5.97 – 5.95	0.997
Race [Black or African Am.]	-3.90	-8.46 – 0.66	0.093
Race [More than one]	5.62	-4.06 – 15.29	0.255
Race [Unknown or Not Reported]	-2.76	-13.96 – 8.45	0.630
Sex [M]	0.95	-1.98 – 3.88	0.523
Income	-0.42	-1.13 – 0.30	0.251
Alcohol Z	0.94	-2.01 – 3.90	0.532
Tobacco Z	-0.68	-2.61 – 1.25	0.490
**Random Effects**			
σ^2	462.86		
$\tau_{00\ \text{Family Structure}}$	29.06		
ICC	0.06		
$N_{\text{Family Structure}}$	434		
Observations	998		
Marginal R^2 / Conditional R^2	0.044 / 0.10		

© 2025 Gowin JL et al. *JAMA Network Open.*

eTable 16. Full Model Examining the Effect of Cannabis Use (History, Recent Use) and Dependence Diagnosis on Activation of the Dorsolateral Prefrontal Cortex (i6-8) During the Working Memory Task

Lifetime history considers number of cannabis uses with levels for 0-10, 11-999, and 1000+ entered as ordered factors (levels 0, 1, 2). Linear and quadratic effects are fit. Dependence diagnosis is based on DSM-IV criteria. THC indicates recent use. For race, the reference group is white. For 1st use (of cannabis), the reference group is "Never". The p-values are uncorrected.

	**Dorsolateral PFC during Working Memory**		
Predictors	*Estimates*	*CI*	*p*
(Intercept)	2.01	-12.14 – 16.16	0.781
Lifetime History [linear]	-4.99	-10.28 – 0.30	0.065
Lifetime History [quadratic]	-4.45	-8.02 – -0.88	**0.015**
THC [Positive]	-4.99	-10.52 – 0.54	0.077
Dependence Dx [Yes]	2.14	-3.94 – 8.22	0.490
1st Use <14	-0.24	-7.80 – 7.32	0.950
1st Use 15-17	0.71	-3.95 – 5.36	0.766
1st Use 18-20	0.29	-3.96 – 4.54	0.895
1st Use 21+	-1.53	-6.35 – 3.28	0.532
Education	2.23	1.32 – 3.13	**<0.001**
Race [Am. Indian/Alaskan Nat.]	-0.84	-31.25 – 29.57	0.957
Race [Asian/Nat. Hawaiian/Othr Pacific Is.]	1.60	-4.65 – 7.85	0.616
Race [Black or African Am.]	-6.48	-11.32 – -1.64	**0.009**
Race [More than one]	8.73	-1.11 – 18.56	0.082
Race [Unknown or Not Reported]	0.61	-10.63 – 11.85	0.915
Sex [M]	2.50	-0.46 – 5.46	0.098
Income	-0.01	-0.72 – 0.69	0.967
Alcohol Z	-0.44	-3.42 – 2.54	0.771
Tobacco Z	-0.42	-2.36 – 1.51	0.667
**Random Effects**			
σ^2	410.89		
τ_{00} Family Structure	85.15		
ICC	0.17		
N Family Structure	434		
Observations	998		
Marginal R^2 / Conditional R^2	0.081 / 0.238		

© 2025 Gowin JL et al. *JAMA Network Open.*

eTable 17. Full Model Examining the Effect of Cannabis Use (History, Recent Use) and Dependence Diagnosis on Behavioral Accuracy During the Working Memory Task

Lifetime history considers number of cannabis uses with levels for 0-10, 11-999, and 1000+ entered as ordered factors (levels 0, 1, 2). Linear and quadratic effects are fit. Dependence diagnosis is based on DSM-IV criteria. THC indicates recent use. For race, the reference group is white. For 1st use (of cannabis), the reference group is “Never”. The p-values are uncorrected.

	**Working Memory Task Accuracy**		
Predictors	*Estimates*	*CI*	*p*
(Intercept)	70.08	64.75 – 75.40	**<0.001**
Lifetime History [linear]	0.54	-1.43 – 2.50	0.591
Lifetime History [quadratic]	-0.90	-2.22 – 0.42	0.182
THC [Positive]	-2.29	-4.35 – -0.24	**0.029**
Dependence Dx [Yes]	0.68	-1.56 – 2.93	0.551
1st Use <14	-1.53	-4.37 – 1.31	0.291
1st Use 15-17	-0.96	-2.69 – 0.78	0.280
1st Use 18-20	-0.29	-1.87 – 1.29	0.721
1st Use 21+	-0.80	-2.58 – 0.99	0.381
Education	1.16	0.82 – 1.50	**<0.001**
Race [Am. Indian/Alaskan Nat.]	-2.23	-13.34 – 8.89	0.695
Race [Asian/Nat. Hawaiian/Othr Pacific Is.]	1.70	-0.75 – 4.14	0.174
Race [Black or African Am.]	-4.54	-6.46 – -2.63	**<0.001**
Race [More than one]	1.05	-2.67 – 4.77	0.580
Race [Unknown or Not Reported]	-6.46	-10.66 – -2.25	**0.003**
Sex [M]	2.47	1.36 – 3.58	**<0.001**
Income	-0.00	-0.27 – 0.26	0.977
Alcohol Z	0.40	-0.72 – 1.52	0.482
Tobacco Z	-0.21	-0.93 – 0.51	0.566
**Random Effects**			
σ^2	52.04		
$\tau_{00\ \text{Family Structure}}$	19.48		
ICC	0.27		
$N_{\text{Family Structure}}$	434		
Observations	997		
Marginal R^2 / Conditional R^2	0.153 / 0.383		

© 2025 Gowin JL et al. *JAMA Network Open.*

eTable 18. Full Model Examining the Effect of Cannabis Use (History, Recent Use) and Dependence Diagnosis on Behavioral Accuracy During the Episodic Verbal Learning/Memory Task (Penn Word Task), Indexed as the Total Number of Accurate Response Out of 40

Lifetime history considers number of cannabis uses with levels for 0-10, 11-999, and 1000+ entered as ordered factors (levels 0, 1, 2). Linear and quadratic effects are fit. Dependence diagnosis is based on DSM-IV criteria. THC indicates recent use. For race, the reference group is white. For 1st use (of cannabis), the reference group is "Never". The p-values are uncorrected.

	**Episodic Verbal Memory**		
Predictors	*Estimates*	*CI*	*p*
(Intercept)	33.03	31.20 – 34.86	**<0.001**
Lifetime History [linear]	0.01	-0.67 – 0.69	0.981
Lifetime History [quadratic]	-0.28	-0.74 – 0.18	0.231
THC [Positive]	-0.86	-1.57 – -0.14	**0.019**
Dependence Dx [Yes]	-0.08	-0.86 – 0.71	0.849
1st Use <14	-0.00	-0.98 – 0.97	0.995
1st Use 15-17	0.05	-0.55 – 0.65	0.877
1st Use 18-20	0.08	-0.47 – 0.63	0.780
1st Use 21+	-0.13	-0.75 – 0.50	0.692
Education	0.18	0.07 – 0.30	**0.002**
Race [Am. Indian/Alaskan Nat.]	-2.28	-6.19 – 1.64	0.254
Race [Asian/Nat. Hawaiian/Othr Pacific Is.]	1.00	0.18 – 1.83	**0.017**
Race [Black or African Am.]	-0.06	-0.69 – 0.57	0.861
Race [More than one]	0.89	-0.38 – 2.16	0.171
Race [Unknown or Not Reported]	-0.01	-1.46 – 1.44	0.991
Sex [M]	-0.42	-0.81 – -0.04	**0.030**
Income	0.03	-0.06 – 0.12	0.566
Alcohol Z	-0.22	-0.61 – 0.17	0.263
Tobacco Z	-0.05	-0.30 – 0.20	0.675
**Random Effects**			
σ^2	6.74		
$\tau_{00\ \text{Family Structure}}$	1.56		
ICC	0.19		
$N_{\text{Family Structure}}$	433		
Observations	995		
Marginal R^2 / Conditional R^2	0.055 / 0.233		

© 2025 Gowin JL et al. *JAMA Network Open.*

eTable 19. Full Model Examining the Effect of Cannabis Use (History, Recent Use) and Dependence Diagnosis on Behavioral Accuracy During the Theory of Mind Task, Indexed by the Percentage of Correct Identifications of Social Interactions

Lifetime history considers number of cannabis uses with levels for 0-10, 11-999, and 1000+ entered as ordered factors (levels 0, 1, 2). Linear and quadratic effects are fit. Dependence diagnosis is based on DSM-IV criteria. THC indicates recent use. For race, the reference group is white. For 1st use (of cannabis), the reference group is “Never”. The p-values are uncorrected.

	**Theory of Mind Behavioral Accuracy**		
Predictors	*Estimates*	*CI*	*p*
(Intercept)	77.33	69.72 – 84.94	**<0.001**
Lifetime History [linear]	-0.59	-3.45 – 2.26	0.683
Lifetime History [quadratic]	-0.55	-2.48 – 1.38	0.575
THC [Positive]	-3.21	-6.21 – -0.22	**0.035**
Dependence Dx [Yes]	2.20	-1.08 – 5.49	0.188
1st Use <14	-0.03	-4.10 – 4.03	0.987
1st Use 15-17	-0.13	-2.64 – 2.38	0.920
1st Use 18-20	-0.59	-2.88 – 1.70	0.612
1st Use 21+	1.03	-1.57 – 3.63	0.437
Education	0.97	0.48 – 1.46	**<0.001**
Race [Am. Indian/Alaskan Nat.]	-30.37	-46.81 – -13.93	**<0.001**
Race [Asian/Nat. Hawaiian/Othr Pacific Is.]	3.09	-0.27 – 6.44	0.072
Race [Black or African Am.]	-4.22	-6.80 – -1.64	**0.001**
Race [More than one]	1.84	-3.44 – 7.12	0.494
Race [Unknown or Not Reported]	2.01	-4.03 – 8.06	0.514
Sex [M]	2.61	1.02 – 4.21	**0.001**
Income	0.29	-0.09 – 0.67	0.140
Alcohol Z	-0.65	-2.26 – 0.95	0.425
Tobacco Z	-0.19	-1.24 – 0.85	0.718
**Random Effects**			
σ^2	121.71		
τ_{00} Family Structure	21.56		
ICC	0.15		
N Family Structure	434		
Observations	997		
Marginal R^2 / Conditional R^2	0.098 / 0.234		

© 2025 Gowin JL et al. *JAMA Network Open.*

eFigure 1. Effects of Diagnosis of Cannabis Dependence on Average Activation During Each of the 7 Tasks

The effect size was estimated by comparing individuals with a history (N= 93) of cannabis dependence versus no history (N = 912) of cannabis dependence. None of the tasks showed a significant effect of diagnosis.

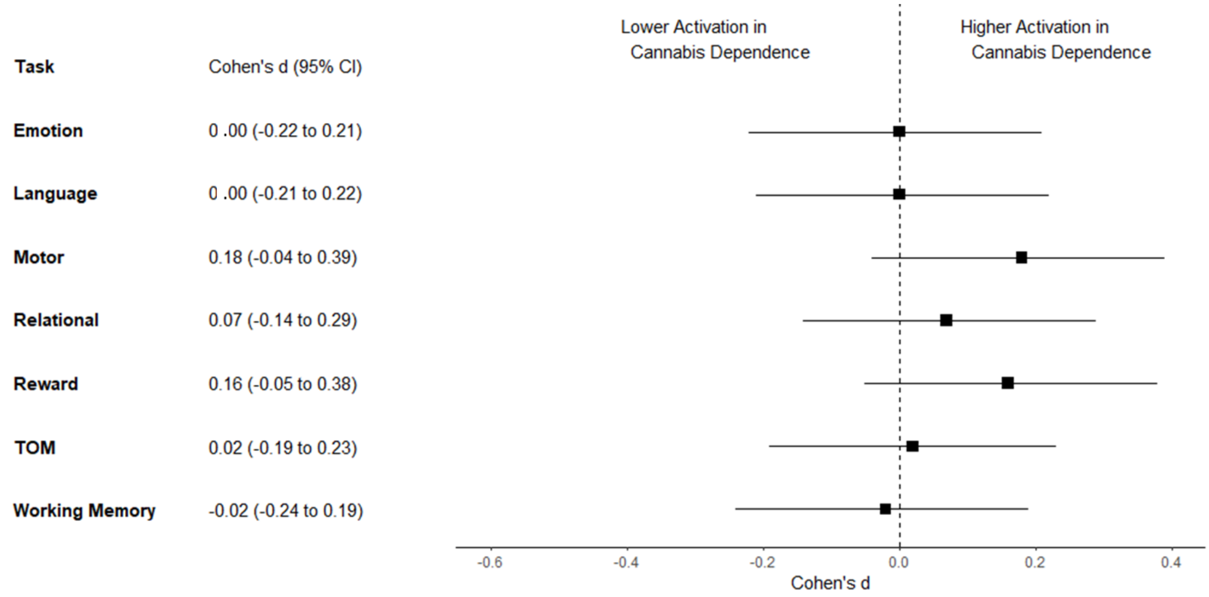

© 2025 Gowin JL et al. *JAMA Network Open.*

eFigure 2. Effects of Lifetime History of Cannabis Use on Average Activation During Each of the 7 Tasks

The effect size was estimated by comparing lifetime moderate users (N = 179) to non-users (N=736). Lifetime moderate users were defined by having used cannabis between 10 and 999 times. Non-users were defined by <10 lifetime uses of cannabis. None of the tasks showed a significant effect.

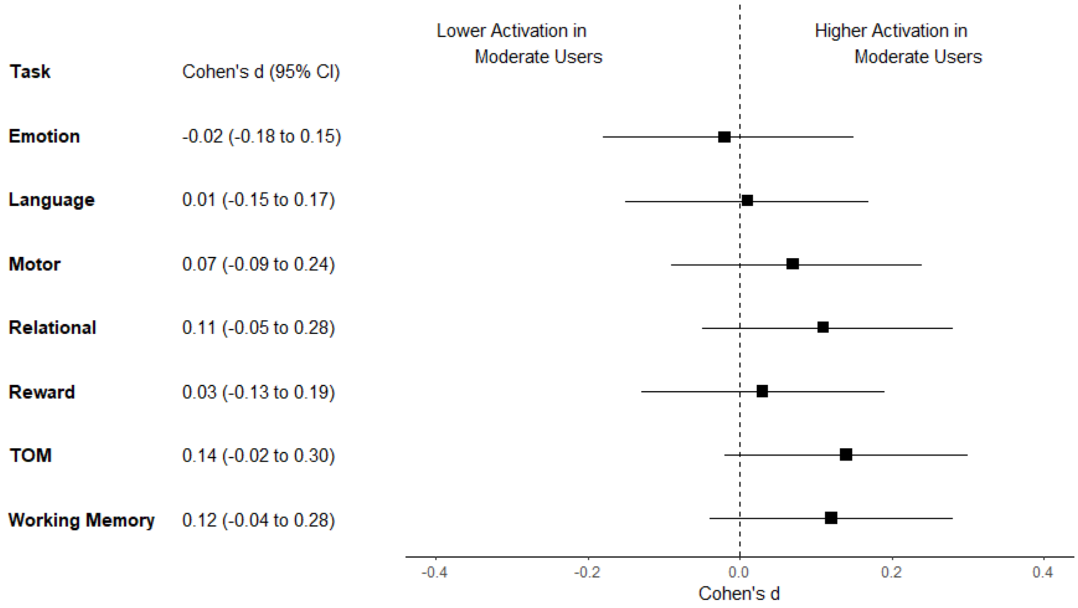

© 2025 Gowin JL et al. *JAMA Network Open.*

eFigure 3. Effects of Lifetime History of Cannabis Use on Average Activation During Each of the 7 Tasks

The effect size was estimated by comparing lifetime moderate users (N = 179) to lifetime heavy users (N = 88). Lifetime heavy users were defined as having used cannabis over 1000 times. Moderate users were defined by having used cannabis between 10 and 999 times.

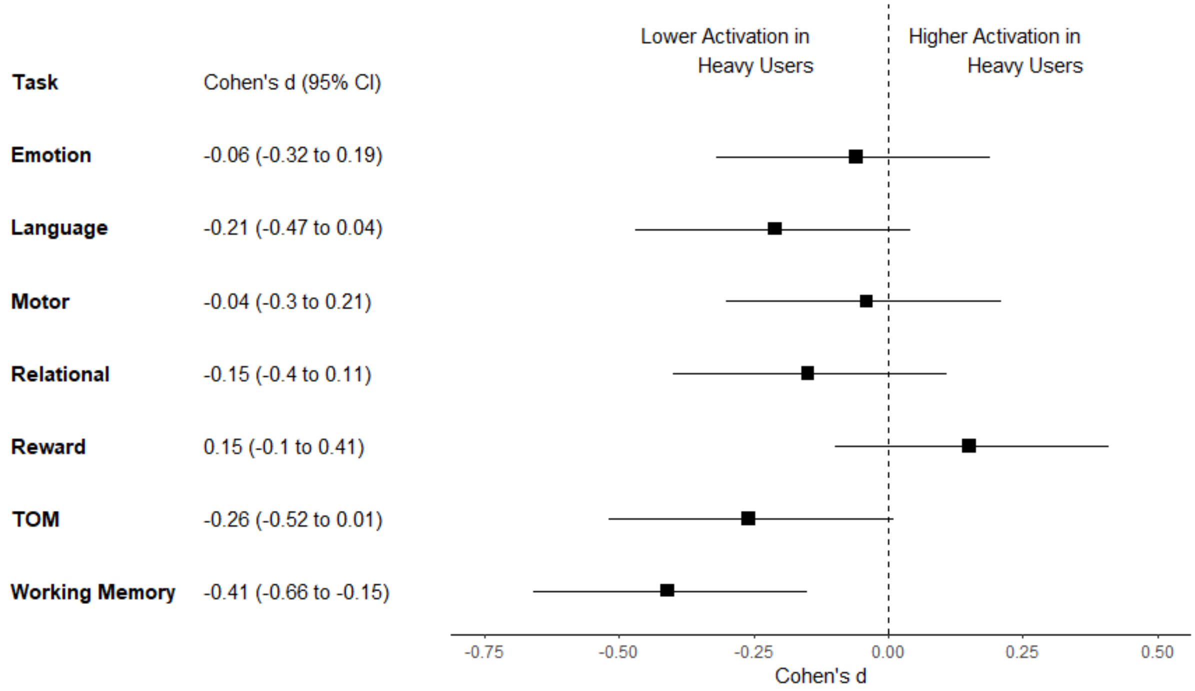

© 2025 Gowin JL et al. *JAMA Network Open.*

eFigure 4. Correlation Matrix Showing the Relationship Between Verbal Episodic Memory and Crystallized Intelligence With Income, Education, and the Average Activation From the 7 Tasks

Values represent the Spearman's correlation coefficient (ρ). Intelligence was moderately associated with activation from the language, working memory, theory of mind, and relational task. Red outline indicates $p < 0.001$.

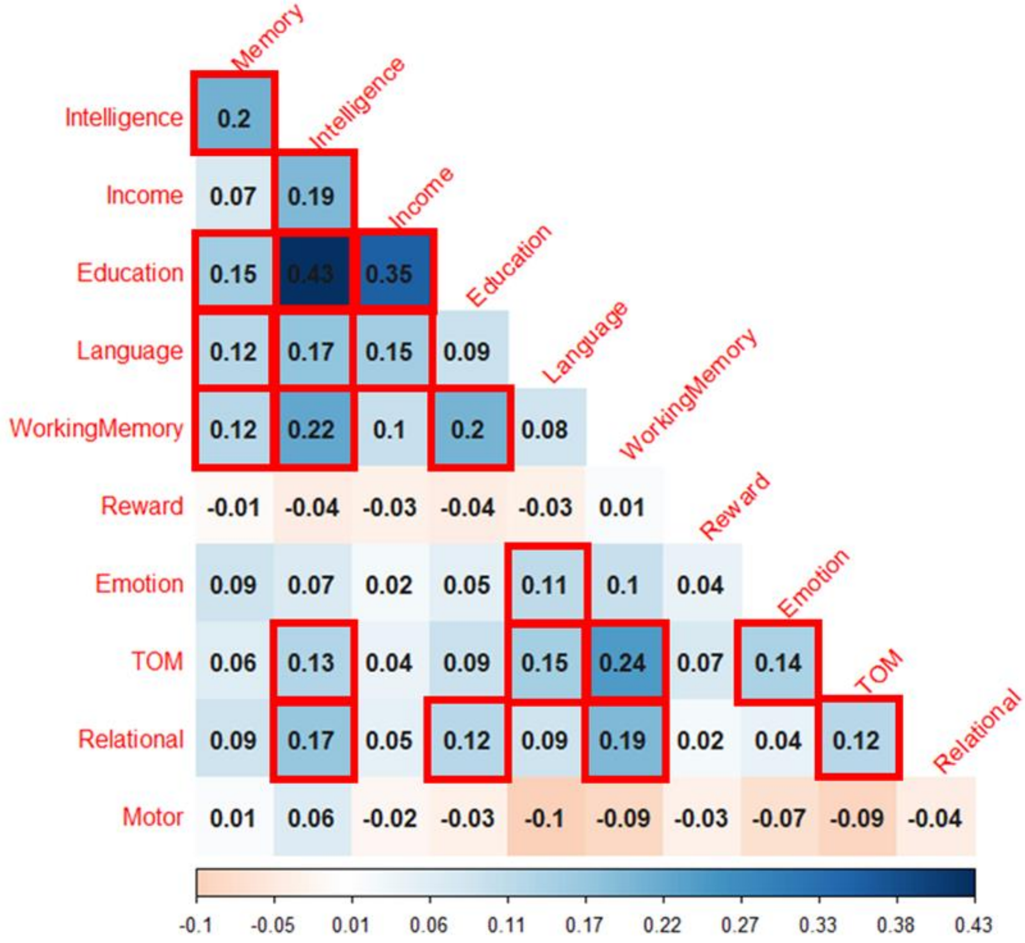

© 2025 Gowin JL et al. *JAMA Network Open.*

eFigure 5. Sex-by-Tetrahydrocannabinol (THC) Interaction During the Motor Task

Tukey's post-hoc testing revealed that males who were positive for THC (N= 74) showed lower activation during the task compared to males who were negative for THC (N = 397) with an adjusted *p* = 0.01. Post-hoc testing revealed that females who were positive for THC (N = 32) did not differ from females who were negative for THC (N = 502) with an adjusted *p* = 0.24.

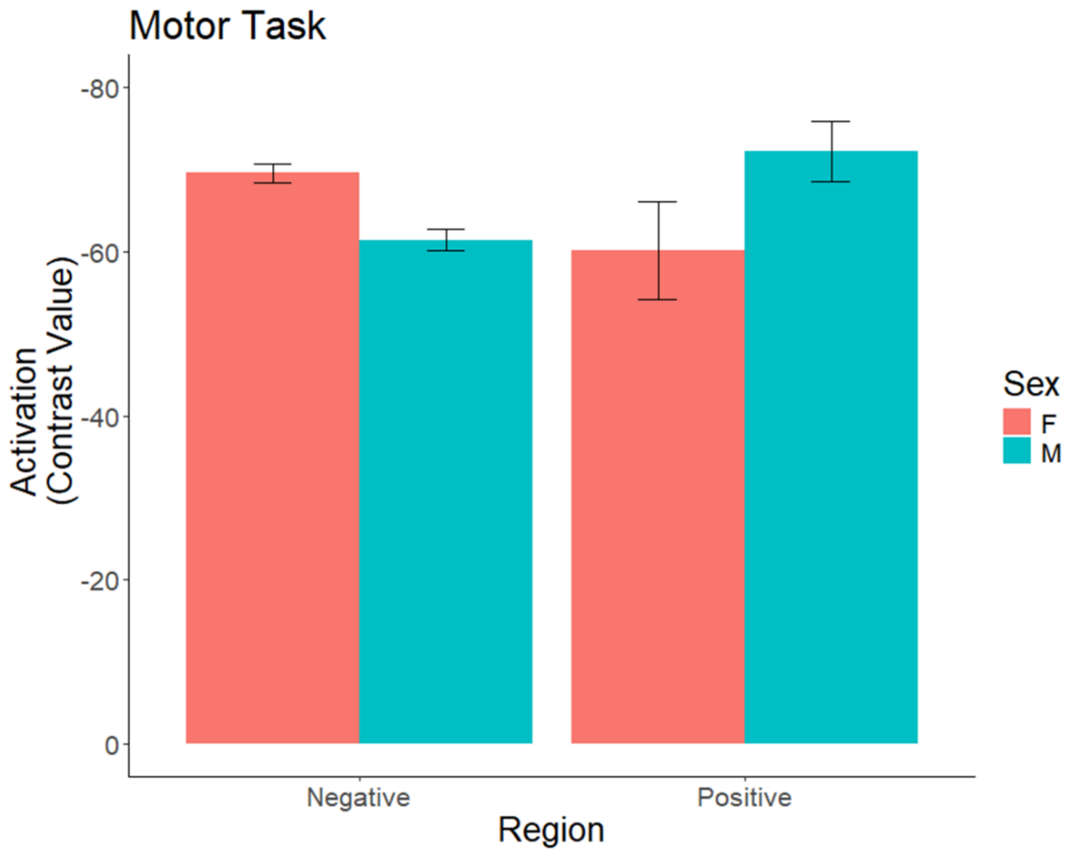

© 2025 Gowin JL et al. *JAMA Network Open.*

eReferences

1. Glasser MF, Coalson TS, Robinson EC, et al. A multi-modal parcellation of human cerebral cortex. *Nature*. 2016/08/01 2016;536(7615):171-178. doi:10.1038/nature18933
2. Hariri AR, Mattay VS, Tessitore A, et al. Serotonin transporter genetic variation and the response of the human amygdala. *Science*. 2002;297(5580):400-403.
3. Delgado MR, Nystrom LE, Fissell C, Noll DC, Fiez JA. Tracking the hemodynamic responses to reward and punishment in the striatum. *Journal of neurophysiology*. 2000;84(6):3072-3077.
4. Binder JR, Gross WL, Allendorfer JB, et al. Mapping anterior temporal lobe language areas with fMRI: a multicenter normative study. *Neuroimage*. 2011;54(2):1465-1475.
5. Buckner RL, Krienen FM, Castellanos A, Diaz JC, Yeo BTT. The organization of the human cerebellum estimated by intrinsic functional connectivity. *Journal of neurophysiology*. 2011;106(5):2322-2345.
6. Yeo BTT, Krienen FM, Sepulcre J, et al. The organization of the human cerebral cortex estimated by intrinsic functional connectivity. *Journal of neurophysiology*. 2011;
7. Smith R, Keramatian K, Christoff K. Localizing the rostrolateral prefrontal cortex at the individual level. *Neuroimage*. 2007;36(4):1387-1396.
8. Thompson RKR, Oden DL, Boysen ST. Language-naive chimpanzees (Pan troglodytes) judge relations between relations in a conceptual matching-to-sample task. *Journal of Experimental Psychology: Animal Behavior Processes*. 1997;23(1):31.
9. Christoff K, Prabhakaran V, Dorfman J, Zhao Z, Kroger JK, Holyoak KJ, Gabrieli JDE. Rostrolateral prefrontal cortex involvement in relational integration during reasoning. *Neuroimage*. 2001;14(5):1136-1149.
10. Wheatley T, Milleville SC, Martin A. Understanding animate agents: distinct roles for the social network and mirror system. *Psychological science*. 2007;18(6):469-474.
11. Abell F, Happe F, Frith U. Do triangles play tricks? Attribution of mental states to animated shapes in normal and abnormal development. *Cognitive Development*. 2000;15(1):1-16.
12. Miller MB, Donovan C-L, Van Horn JD, German E, Sokol-Hessner P, Wolford GL. Unique and persistent individual patterns of brain activity across different memory retrieval tasks. *Neuroimage*. 2009;48(3):625-635.
13. Barch DM, Burgess GC, Harms MP, et al. Function in the human connectome: task-fMRI and individual differences in behavior. *Neuroimage*. 2013;80:169-189.

© 2025 Gowin JL et al. *JAMA Network Open.*
