# Supplementary material for: Brain Function Outcomes of Recent and Lifetime Cannabis Use
Source: JAMA Netw Open. 2025 Jan 28;8(1):e2457069. doi: 10.1001/jamanetworkopen.2024.57069 (PMC11775743; doi:10.1001/jamanetworkopen.2024.57069)
Supplement: Supplement 2. — Data Sharing Statement [file jamanetwopen-e2457069-s002.pdf]

Data Sharing Statement

Gowin. Brain Function Outcomes of Recent and Lifetime Cannabis Use. *JAMA Netw Open*. Published January 28, 2025. doi:10.1001/jamanetworkopen.2024.57069

Data
Data available: No

Additional Information
Explanation for why data not available: The data is publicly available via the Human Connectome Project
